# Collaborative software for traditional and translational research

**DOI:** 10.1186/1479-7364-6-21

**Published:** 2012-09-25

**Authors:** Ari E Berman, William K Barnett, Sean D Mooney

**Affiliations:** 1 Buck Institute for Research on Aging, Bioinformatics Core, 8001 Redwood Blvd, Novato, CA, 94945, USA; 2 Indiana University, Bloomington, IN, 47405, USA

## Abstract

Biomedical research has entered a period of renewed vigor with the introduction and rapid development of genomic technologies and next-generation sequencing methods. This research paradigm produces extremely large datasets that are both difficult to store and challenging to mine for relevant data. Additionally, the thorough exploration of such datasets requires more resources, personnel, and multidisciplinary expertise to properly analyze and interpret the data. As a result, modern biomedical research practices are increasingly designed to include multi-laboratory collaborations that effectively distribute the scientific workload and expand the pool of expertise within a project. The scope of biomedical research is further complicated by increased efforts in translational research, which mandates the translation of basic laboratory research results into the human medical application space, adding to the complexity of potential collaborations. This increase in multidisciplinary, multi-laboratory, and biomedical translational research identifies a specific need for formalized collaboration practices and software applications that support such efforts. Here, we describe formal technological requirements for such efforts and we review several software solutions that can effectively improve the organization, communication, and formalization of collaborations in biomedical research today.

## Introduction

The field of molecular genomics is entering a period of rapid expansion with advancements in next-generation sequencing technologies, clinical sample biobanking and large-scale clinical data integration. The new rapid sequencing techniques allow for the complete sequencing of entire genomes in only a few days and for a fraction of the cost of Sanger sequencing methods [1]. The rate at which this technology is advancing is exceeding the predicted rate of technology development described by Moore's law [2], thus putting the scientific community in the position of being unable to store, process, and ultimately share the large amount of data that comes from these technologies. This new era of genomics, along with the data being generated by older high-throughput technologies (such as genome-wide microarray studies, high-throughput RT-PCR, and large-scale RNAi screens), can produce terabytes of data that will ultimately need to be shared among scientists and clinicians in a collaborative, secure, and translational manner as part of a research program. Thus, effective mechanisms for the sharing, or even storing these data, are unclear at best. Currently, scientists find themselves at a crossroads of scientific technology advancement that requires highly multidisciplinary and collaborative research designs.

As the rate of data accumulation increases, the complexity of both research design and execution is also increasing [3]. Basic research projects require ever-increasing amounts of detail, while clinical studies require greater numbers in their cohorts as well as multidisciplinary approaches, leaving the efforts of bridging the two research areas in a doubly troubled state. Currently, effective research requires more resources and more people. Since the resources of individual labs are somewhat limited, this usually results in collaborations with other laboratories with specific expertise in various portions of the research design, thus improving the effectiveness, and overall quality of the research being performed, but at greater risk due to the inherent interdependencies between research groups. In order to support this type of research, a highly advanced data infrastructure is required. The combination of the data management problem, the increasing number of collaborations, and the complexity of research projects presents an emergent problem that requires high-capacity data storage and the ability to effectively share those data at a distance in order to collaborate effectively.

With the continued refinement of next-generation sequencing technologies, many clinical studies are incorporating advanced genomics techniques into their data collection sets, resulting in large collections of human sequence information and their associations with human phenotypes. The richness of these sequences, as well as the accompanying human data, is of great value to the basic research community. Additionally, the application of basic research findings to the clinical arena through translational research is critically important. Currently, much of the data produced by basic research never find its way into clinical practice, and very little of the data generated by clinical researchers is incorporated into basic research design in the laboratory [4]. To help solve this problem, the National Institutes of Health has initiated a program to promote translational research called the Clinical and Translational Science Awards (CTSA) [5,6]. The CTSA program is intended to develop educational and research infrastructures at academic institutions and is charged with creating a national collaborative consortium to drive biomedical research towards therapies [7], including the promotion of collaborative sharing of data across translational lines through the development of online resources aimed towards accomplishing this goal.

There are many existing software packages that enable research collaboration, and the use of such tools by basic and clinical investigators would improve the effectiveness of both data sharing and workflows among collaborators. It is important to note, however, that these systems do not solve the data deluge problem that the scientific community is currently facing. The storage capacity of many of these systems is scalable, but the transfer of large amounts of data over the internet remains a problem. Here, we suggest a more optimized organization of collaboration. Rather than exchanging raw data among collaborators, a carefully organized collaboration effort, coordinated by an accessible, online collaboration software solution, would require only that pre-analyzed data be shared across distances in most cases. This paradigm drastically reduces the amount of data that requires sharing and makes centralized organization via online loci a viable mid-level solution for collaboration in the current era of human genomics research. Here, we review some of the most used and some up-and-coming collaborative systems and compare their effectiveness for wide-scale distribution of data and information on the level of basic biomedical research.

## Software that enables group collaboration

For the purposes of this review, we chose several existing web-based collaboration systems that are in the widest use in science. Inclusion of tools used in this review was based on systems known to be currently in use at medical centers or systems known to be under development specifically for the use in translational biomedical research. Systems included in this review article are Alfresco [8], Confluence [9], Basecamp [10], SharePoint [11], HUBzero [12], Laboratree [13], and MediaWiki [14].

The available features, accessibility, user experience, and effectiveness for scientific collaboration were qualitatively assessed for each of the systems named above. Each system was either installed locally, or subscribed to, and used in a real-world setting of collaborative research at the Buck Institute for Research on Aging. The features of each system and how they compare to each other were summarized in Table 1. The features were compared through a combination of synthesizing the existing feature lists from each tool's website and through actual use of the product. Features were broken down into several feature categories, as shown in Table 1.

**Table 1:** Comparison of features of collaborative tools

**Features**	**Alfresco**	**Confluence**	**Basecamp**	**SharePoint**	**Laboratree**	**MediaWiki**	**HUBzero**
*Document management*							
Storage/retrieval	X	X	X	X	X	X	X
Version control	X	X	X	X	X		
Audit	X						
Search	X	X		X	X	X	X
Categorize files (tags)	X	X	X	X	X		X
Sort	X	X	X	X	X		X
Customizable views				X			
Office/SharePoint integration	X			X			
Online presentations							X
*Information management*							
Calendar				X	X		X
Contacts			X	X			
Blogs	X				X		X
Discussions	X	X	X	X	X	X	X
Wikis	X	X			X	X	X
News feeds	X	X			X	X	X
Issues				X			
Lists		X	X	X	X		
Announcements		X		X	X		X
Email notifications		X			X	X	X
Interactive online tools							X
*Collaboration*							
User-based access control		X		X		X	
Social networking-based access control	X		X		X		X
Static project workspaces	X	X	X		X		X
Customizable project workspaces				X		X	
Project-specific discussions	X	X		X	X	X	X
Surveys				X			
Task lists			X	X	X		X
Customizable workflows	X	X^a^		X		X^a^	X
Instant messaging					X		
*Interface*							
Web-based: CMS-like	X	X	X	X	X		X
Web-based: HTML or pseudo-code						X	
Windows share (CIFS)	X	X		X			
FTP				X			
WebDAV	X			X		X^a^	X
Extensible using plugins			X			X	X
*Installation*							
Centrally hosted: web-only		X	X		X		X
Locally hosted: web	X	X		X	X	X	X
Locally hosted: GUI							
Local install	X	X		X	X	X	X
Linux	X	X			X	X	X
Windows	X	X		X		X	
Macintosh	X	X				X	
Virtual machine							X
*License*							
Subscription fee (mandatory)		X	X				
Subscription fee (enterprise, optional)	X						X
Software purchase				X			
License contract		X		X	X		
Open source (free)	X					X	X
Closed source (free)					X		

Each of the features is divided into feature sets, and an inclusion of a feature in a particular collaboration solution is indicated with an 'X' mark below that piece of software; ^a^available by extension.

**Figure 1:**
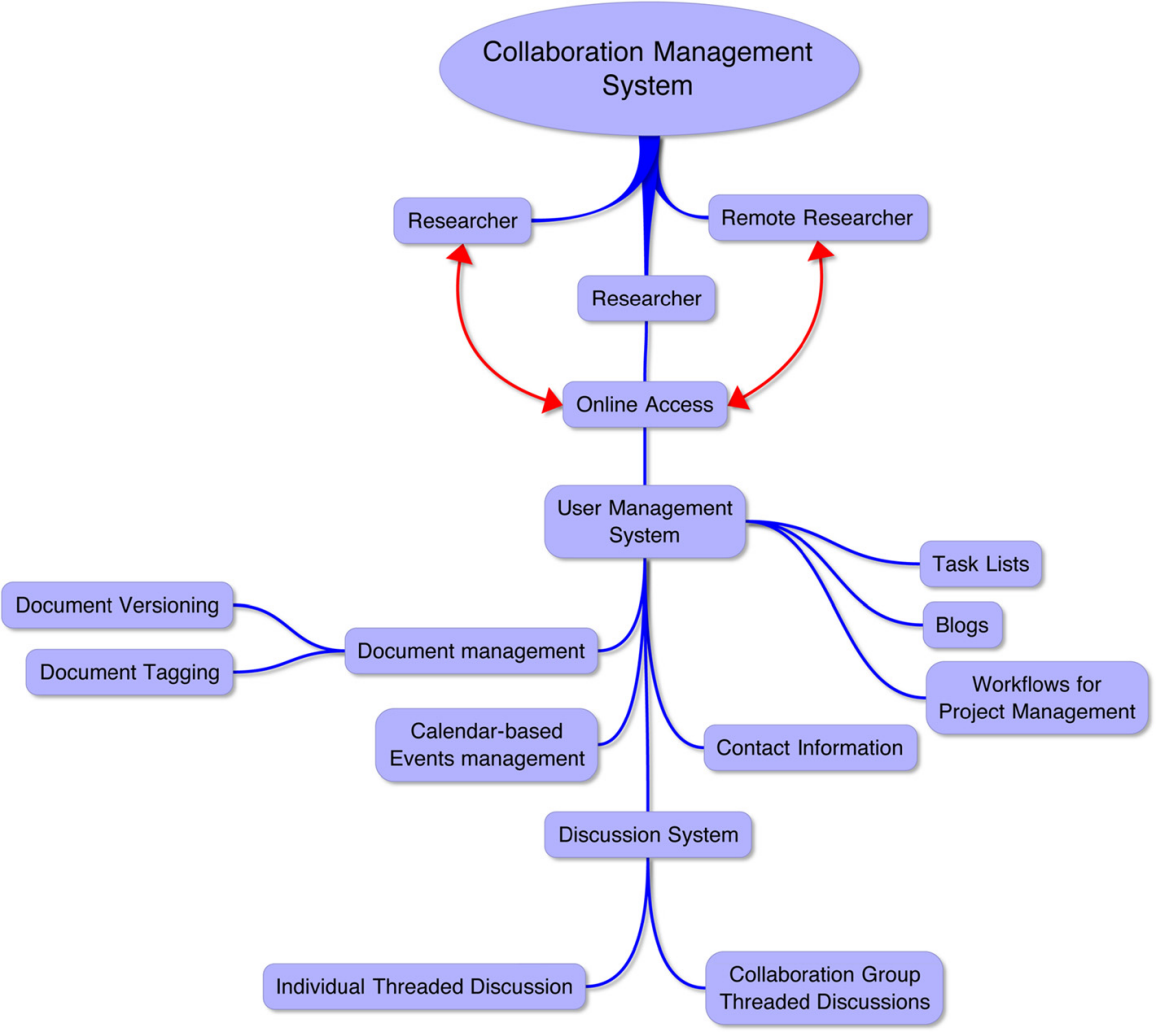
**Graphical representation of the basic functions that collaborative software solutions must maintain.** The figure shows the basic workflow and general relationships of software functionality and how they relate to researchers' user experience. This workflow shows a user-centric view of collaboration and could be indicative of a social networking-based collaboration solution.

The tools reviewed in this article all provide basic collaborative functionality to varying degrees. Though many of the commercially developed systems were designed for use in corporate environments rather than scientific settings, the tools all focus on the common challenges of collaborative information management. In general, features of these tools should include document management, coordination of groups of users, common access points, event coordination, and discussions (Figure 1). More specific features that might be helpful to translational research environments include document versioning and tagging, public vs. private datasets, contact information, workflows for post-processing data, blogs and discussions common to collaborative groups, user-configurable email notifications, and task lists (Figure 1). Additionally, features that would improve collaboration discovery involve advanced social networking functions (e.g., suggesting linkages based on user profile information), public searches that browse concept tagging and categories of private data and projects, and user-friendly interfaces with easy-to-understand functionality. Each of the reviewed collaborative tools and their features are discussed below.

### Alfresco

The Alfresco CMS collaboration system is developed and maintained by the Alfresco Development Group [8]. The company offers their collaboration system in both hosted and local versions. The hosted solution is available as a cloud server environment so that deployment does not require any actual local hardware or networking expertise. The locally installed solutions include Alfresco Enterprise Edition and Alfresco Community Edition. The Community Edition is free for use as a download and comes with no support, other than developer support in the forums. An Enterprise subscription entitles users to technical support, knowledge base support, installation and configuration assistance, a warranty, and supported software maintenance. The Alfresco software is open source and developed in Java. The system operates through the Tomcat Java web server, with Alfresco running as a servlet under this system. As such, the packaged installer will operate relatively seamlessly on any operating system.

Alfresco's major features (summarized in Table 1) include customizable collaboration sites, event calendars, site-specific wikis, blogs, document management, links, forum-style (threaded) discussions, and lists, although its greatest strength is in document management. Each user has their own space to store documents and information specific to them, and each user may create their own collaboration sites within the Alfresco system-each site representing a specific research project. From there, users can easily invite existing or new users to the site and assign an access level to them. Access levels include Site Manager, Collaborator, Contributor, and Consumer, with the implied access rights for each. Managers can also create user groups to more elegantly define user access rights within a site. All access levels, except Consumer, can add documents and join in discussions, and collaborators have full access to the sites' functions, short of user administration.

Each collaboration site has a customizable dashboard as well as customizable pages associated with it. The dashboard provides a mechanism for rapid access to the most important features on the site. Each collaboration site also has a self-contained wiki (user-contributed content) that can be used to provide static documentation, notes, protocols, and shared information. Additionally, a specific wiki entry can be featured on the dashboard for informational purposes. A site-specific blog can also be utilized to update collaborators of recent developments or to rehash recent events, while the discussion section can be used for online conversations regarding collaboration-related business. Links and calendars can be used to organize deadlines, events, and important online information for the site, and data lists can be used to assign workflows, tasks, and deadlines to the members of the site. Additionally, Alfresco Share provides a document management system that accepts most common document types, tracks version information, supports document checkout and check-in, and provides on-screen document viewing, metadata, tags, comments, and sharing. In addition, the document library can be accessed via Windows shares, WebDAV, and NFS for ease of use. All of the described features are accessible via a web-based Java servlet interface that is interactive, user-friendly, and logical. Alfresco Share's combination of features and interface provides a collaborative environment suitable for a variety of applications in translational medicine and basic science.

### Confluence

The Confluence collaboration system is a wiki-based collaboration management system developed by the Australian-based company Atlassian. The software utilizes online authoring solutions, Microsoft Office and SharePoint integration, and a plugin catalog to create custom collaborative environments for a wide array of potential users. Similar to Alfresco, Confluence is available in both hosted and locally installable forms. Both solutions are available under a 'number of users'-based pricing structure; however, the software is free to nonprofit organizations. Confluence is developed as a closed-source system, with many open-source components within its structure. The interface is web-based and runs as a Java servlet on a Tomcat server, much like the Alfresco system. The supported database backend is MySQL, PostgreSQL, or Oracle. Atlassian additionally offers a hosted solution that can be set up with relative ease and has a slightly different pricing structure. In either case, Confluence is widely used and has many features that are applicable to translational research.

The Confluence collaboration system is based upon wiki technology, and the underlying interface is indicative of this construction. Wikis are websites that allow the easy creation and collaborative editing of any number of interlinked web pages using a simplified markup language. Wikis generally provide a forum for user-contributed and user-maintained content, and thus provide a logical platform from which to create a collaborative software package. The content of a collaboration 'space' is managed via a typical wiki-based editing system. The editor is WSIWYG for the most part, other than the functional macro code that can be inserted into the text being entered to provide various levels of interactivity for that page. Macros can include dynamic content such as lists, tasks, calendars, etc. and can be inserted into a document using an 'insert' dropdown menu listed at the top of the editor interface. Out-of-the-box macros include basic functions such as image galleries, a recently updated content list, task lists, interactive widgets, table of contents, and approximately 70 other macros that are selectable from a pop-up browser in the editing window. The default functionality of Confluence can be enhanced using a plugin repository available from their website that currently holds approximately 300 plugins. Among the immediately useful plugins are attachment check-in/checkout (versioning support for documents) and calendar (not supported for Confluence 3.3). The plugins are installable via the administrative interface and can be configured there as well. The administrative interface imparts extensive control of the system to the administrative user(s), and one can control most aspects of the system, including backups, users, permissions, quotas, etc. *De novo* user registration is not supported out of the box, and an administrator must create new users from the administrative interface. Once active on the system, users can create collaborative spaces, add content or 'child' pages, upload documents to a personal repository, and edit the content of the spaces they have created. Users can be invited to existing spaces by the space managers, and through the use of a plugin, users not currently registered on the system can also be invited to existing spaces. Confluence provides a contained 'intranet' for shared contributions to projects and has a highly extensible feature set that could provide an ideal translational research environment.

### Microsoft SharePoint

SharePoint is a software platform developed by Microsoft for collaboration, web publishing, and file sharing. The software is available in three editions: SharePoint Foundation 2010 (free add-on to Microsoft Server 2008), SharePoint Server 2010 Standard, and Sharepoint Server 2010 Enterprise which are both subject to a licensing fee. Microsoft additionally offers an online solution called SharePoint Online as a cloud service. SharePoint Server provides a number of added features compared to SharePoint Foundation, the majority of which are social networking, efficiency, and advanced metadata classifications and searches. These added features are pertinent to a corporate environment, though some may be useful in academic and translational research. SharePoint Foundation, however, has many features in common with the other collaboration systems reviewed thus far and is the edition that was evaluated for the purposes of this review. SharePoint Foundation, as a Microsoft product, is highly integrated with the Microsoft Windows operating system and allows for seamless integration with many prominent Windows products, making it very simple to use for Windows users. As with the other software packages, SharePoint operates via a web front-end and, beginning with SharePoint 2010, supports most of the popular modern web browsers, though less reliable for Macintosh users.

SharePoint is built upon a template interface that results in a collection of 'Web Parts,' or HTML-driven objects (widgets), that can be collected on a single page to accomplish a layout conducive to the collaboration in question. This interface is tightly bound to other popular Microsoft products and allows integration with packages such as Microsoft Office and Microsoft Outlook. The primary interface method is a web browser, which would seem to maximize cross-platform use. However, until the latest release of SharePoint 2010 (which supports Internet Explorer (IE), Firefox, and Safari), many of the active functions available from within SharePoint were only usable from IE, thus making SharePoint a single platform solution. However, the latest version of SharePoint makes extensive use of AJAX, as opposed to Visual Basic, which improves cross-platform compatibility. Collaboration sites are available for access through WebDAV, allowing for filesystem access to the documents in the collaboration, calendar syncing with Outlook, and other Microsoft product integrations. SharePoint Foundation 2010 has a full range of collaboration features that can be used effectively in a scientific environment.

### Basecamp

The Basecamp collaboration system is a web-based project management solution developed by 37Signals, LLC, and it is available at [10]. Basecamp was developed using Ruby on Rails and is available under several pricing structures that allow for varying numbers of total projects (collaborations), amounts of storage space, and users for your subscription. 37Signals also offers a free version of the software that is limited to two projects and 10Â MB of storage space. Since Basecamp is only offered in a centrally hosted solution, the system may not be suitable for the storage and sharing of large amounts of data among collaborators. However, the availability of the system is high and can be accessed from anywhere in the world over the internet, independent of VPNs or network limitations.

Basecamp offers a full range of features to ease and organize collaborations of all levels of complexity (Table 1). The system has a full-featured, threaded messaging system and a fully integrated comment system that allows comments on any content posted to the collaboration site. Additionally, collaborators can be notified by email about updates to content on any given site. Basecamp also has an integrated file sharing system that can handle most data types, includes automatic preview generation, and keeps track of version information for each file, including the ability to revert to previous versions. Workflow assignments, calendar management, to-do lists, and time-tracking features are also available to make it easier to meet deadlines and accomplish collaborative goals. The system also supports reusable templates for use with multiple projects of the same type. Finally, Basecamp offers open-source plugin development and hundreds of existing plugins that extend the functionality of any collaborative site built on their platform. Basecamp is a complete solution for collaboration and would be ideal for scientific research endeavors.

### Laboratree

Laboratree is a web-based collaborative software solution currently being developed specifically for scientists by Selican Technologies [13]. The software product is based on the original Laboratree system developed at the Indiana University Bioinformatics Core. Laboratree will be made available as a site-installable solution for customers, adding a web service to their local network. This collaborative software will provide a common workspace for collaborating researchers to manage their documents and data, communicate, and analyze results. The technology is based on a social networking environment, which, much like Alfresco, takes a social networking approach to collaborations, thus providing many possibilities for flexible scientific interactions. Like Alfresco, users have the freedom to create collaboration groups, invite new and existing users to them, and have multiple people manage collaborations in various capacities. Laboratree is being developed using CakePHP and AJAX and can thus be run on any web server that supports PHP and has a database connection. The software is currently in an alpha release stage and a pricing structure is currently unavailable, but we were able to evaluate this version for the purposes of this review article.

Laboratree's notable features (TableÂ 1) include a file storage and versioning system that includes selective sharing abilities with collaborators or groups of collaborators. Group managers can set file permissions to be read-only, or updatable by group members, allowing for restricted access to sensitive data. The versioning system allows for groups of people editing the same documents or posting data to keep track of all submitted copies, merge information from simultaneously modified documents, and restore the working copy of a document to a previous version of the document. Additionally, tags can be applied to documents allowing them to be searched by category and, when publically available, searched globally by all users of a Laboratree installation, which may assist in the development of new collaborations. Laboratree will feature a fully interactive messaging system that can utilize email notifications and online discussions, as well as real-time instant messaging and comments on posted content. Laboratree will also include calendar management, blogs, wikis, and discussion threads, thus providing for a complete collaborative solution. In its current form, Laboratree is still awaiting the development of features specific for scientists like VIVO [15] integration and NCBO [16] metadata annotation that will make the usage of this system more useful for scientists than the alternatives mentioned here.

### MediaWiki

MediaWiki is a freely available, open-source wiki software package written in PHP and maintained by mediawiki.org, and a project supported by the Wikimedia Foundation. The software, which can be downloaded from [14], is the same base system that runs the popular Wikipedia website (http://www.wikipedia.org). A wiki is any website that allows the creation and editing of any number of dynamically generated webpages using a simplified web-based interface and a graphical (WYSIWYG) text editor to manipulate content. Wikis are generally populated via community contributions and can be used for everything from documentation to full-featured websites. MediaWiki is not intended to be a collaborative solution *per se*, but the software can be used that way, as exemplified by the Confluence software package reviewed above, which is also based on wiki technology.

Wikis are a useful tool for documentation, simple generation of static content, and information exchange, much like the other collaborative tools reviewed in this article. In addition, there are thousands of plugins that greatly extend the functionality of the base system to allow file sharing, versioning, calendars, workflows, discussions, and comment threads. Each page can also have email notifications set by each user to watch for updates. Thus, with the application of a greater learning curve, MediaWiki can be used as a collaborative system that is highly customizable to the needs of the group running it. Since it is open-source software, it is only user-installable, thus requiring the end-user to supply a hosting solution for the system. MediaWiki, through the use of the proper extensions and some user training, can be used as a collaborative solution in scientific environments.

### HUBzero

HUBzero is a web-based platform for creating websites that support scientific discovery, learning, modeling and simulation, and collaboration [17], and is available from [12]. This system was originally developed by Purdue University in conjunction with the National Science Foundation-sponsored Network for Computational Nanotechnology. It is built upon the popular content management system (CMS) Joomla! with a number of components developed specifically for scientific collaboration pre-installed. It can be downloaded and installed on any local web server. Once installed, menus take you to the various installed components, such as wiki, forums, chat, file sharing, etc. A user-configurable template controls the overall look and feel of the site, and there are thousands of free and commercial templates available for Joomla!/HUBzero.

HUBzero is an open-source, freely available collaboration system that was designed specifically for scientific research, and it includes many of the features outlined in the other solutions reviewed in this article. The default HUBzero installation comes with the ability to create user groups, create wikis and blogs, and maintain a knowledge base, and the ability for users to contribute and share resources, an event calendar, news/events, feedback mechanisms, content tagging, etc. Much like the other systems reviewed here, HUBzero features a built-in CMS and a graphical, web-based administrative interface for ease of installation, customization, and management. Aside from the fact that it is freely available, one main advantage to using HUBzero is that, since it is based on the Joomla! CMS, the interface and usefulness of the system is highly extensible. There are over 10,000 open-source and commercial extensions for Joomla! that can be used to rapidly add almost any functionality. Additionally, it has the ability to incorporate simulation software and other computational tools through its RAPPTURE modeling environment, which can enable collaborative analysis in a central location and also automatically access high-performance computer clusters, Condor flocks, and the Teragrid. This added set of features could be a base platform for the centralization of the storage and analysis of very large datasets in a collaborative and translational manner.

## Discussion

The recent advancement of sequencing technologies in conjunction with the constant improvements to, and the availability of, high-throughput 'omics' research has necessitated a rethinking of how scientists perform research. Most research projects now include genome-wide screens of some sort, and the amount of data and the sheer size of the files associated with these analyses call for a shift in the established paradigm for research practices. Additionally, the availability of these methodologies has further complicated research designs and pushed scientific discovery into a system-level discovery era, both in the laboratory and in the clinical research arenas. This paradigm shift has required researchers to form greater numbers of collaborations and has recently resulted in a more established effort to translate basic research findings towards clinical applications. Equally, the genomic data being collected from clinical research are proving to be useful in the basic research arena and particularly for bioinformatics efforts on both ends of the research spectrum. The combination of these factors has led to the need for more established and formalized methods for information exchange and collaborative organization. This review surveyed several existing technologies, all within a similar, high-availability, web-based software genre, that can support collaborative methodologies and facilitate translational research efforts. While these software packages would help streamline collaboration, they do not currently solve the data deluge and large-file sharing problems.

Many of the software packages reviewed here have been developed with generalized collaborations in mind, rather than for specific scientific applications. However, this generalized collaborative model is as useful for scientific collaboration as business collaboration. Specifically, the coauthoring of manuscripts and grants, the maintenance of protocols or scientific methods, the scheduling of tasks and assignment of workflows to individuals, and the coordination of distant groups of scientists working together to solve problems are all issues that can be solved by general collaboration software packages. Alfresco, Confluence, Basecamp, and SharePoint all offer this functionality, and some of these tools can be customized and/or further developed to include directed scientific applications. Tools that were developed specifically for collaborative research, like Laboratree and HUBzero, have features that are generally more useful to the scientific community, like plugins for citation management, centralized analysis software, etc.

There are several scenarios in which collaborative tools can help to improve the transfer of data between basic research and medicine, thus supporting the efforts of CTSA institutions. In one scenario, a research laboratory might be researching the effectiveness of an experimental drug on a cell culture model of the affected disease, while a medical professional has a patient with this disease under their care and traditional medical interventions are failing. In this situation, the researchers might have stored and annotated their current results in a collaborative system that acts as a central organizing platform for their laboratory. If the system allowed for the marking and tagging of various aspects of the annotations, the medical professional would then be able to search for the patient's disease and find this lab's research. Thus, a collaborative tool's organizing nature would have connected these two groups and possibly formed a new collaboration in which the drug is tested both in the laboratory and in the clinic against the actual disease. The combination of the results of these studies might provide more complete insight into both problems and lead to more directed research and potential medical benefits sooner.

In a second scenario, a clinical trial might be testing the effectiveness of a particular treatment found to work in an animal model of a particular disease. The results of the clinical study are maintained within an online collaborative tool. Simultaneously, a research group finds that it cannot reproduce the data that were originally published in the same area by another group. Searching for answers, the research group finds that a clinical study based on that original research is failing to have any effect in humans. The research group then connects with the clinical trial researchers and a collaboration is formed to find out whether the original mechanism is indeed sound, thus creating a direct link in the common goal of translational medicine and improving the treatment potential of this particular disease.

In a final scenario, the online tools support the formation of collaborations between clinical researchers and basic researchers, allowing for the submission of a joint grant to translate findings from a laboratory into a clinical context. This collaboration is unique from others of its kind, not only because the funding mechanism exists to allow the collaboration to be mutually beneficial, but also that it has a mechanism built in for feedback from the clinic to the lab. This type of cyclic research in translational medicine would likely significantly improve the quality and amount of research performed in this arena and would likely lead to faster and higher quality treatments for diseases in humans.

In order for these scenarios to become reality, several features need to be met by collaborative software. The first requirement is high availability and accessibility. In order for collaborators, both local and distant, to effectively collaborate (or discover new collaborations), the software needs to be accessible by a common, cross-platform, internet-connected interface. The most obvious mechanism for these requirements is the World Wide Web. Web applications have reached similar levels of complexity and interactivity as compiled local user interfaces, thanks to technologies like JavaScript, AJAX, PHP, and Java. The next most obvious implementation needed by these tools is security. User privacy and data security are paramount in biomedical research, especially in clinical studies. Collaborative software must be able to support the most intensive security requirements to protect the data and research plans contained within its systems. Conversely, it is also necessary for the tags marking the information contained within these systems to be available for searching from registered users and nonusers alike. Thus, it is important for data contained within the collaborative software to tag and annotate its stored information in a manner that connects searches with research endeavors. Finally, it is vitally important that these systems contain effective file storage and organization systems.

Many of the software solutions reviewed here can accomplish these goals and have already been implemented at CTSA institutions for these purposes. Extending the usage of these tools to the general research population can only further improve this effort and will help scientists to deal with the reality of the constantly increasing need for collaborative efforts in the modern genomics era. Armed with the proper tools and standardized collaboration practices, tools such as these can help drive research forward by simplifying researcher interactions and organizing efforts at a fundamental level, allowing researchers to concentrate on their research.

## Competing interests

The authors would like to declare a conflict of interest in the review of Laboratree, as S.D. Mooney is the Consulting Director of Product Development for Selican Technologies, Inc. Laboratree was reviewed and written up by A.E. Berman, who is not affiliated with Selican Technologies in any way.

## Authors' contributions

AB performed all comparative software installation, testing, and comparisons as well as drafted the manuscript. SM provided editing feedback, and SM and WB provided contextual feedback for the software compared in the manuscript. All authors read and approved the final manuscript.
